# MicroRNA-410 Reduces the Expression of Vascular Endothelial Growth Factor and Inhibits Oxygen-Induced Retinal Neovascularization

**DOI:** 10.1371/journal.pone.0095665

**Published:** 2014-04-28

**Authors:** Na Chen, Jiaqi Wang, Yijun Hu, Bei Cui, Wenjie Li, Guixia Xu, Lin Liu, Shanrong Liu

**Affiliations:** 1 Department of Ophthalmology, Ren Ji Hospital, Shanghai Jiao Tong University School of Medicine, Shanghai, China; 2 Department of Ophthalmology, First Affiliated Hospital of Second Military Medical University, Shanghai, China; 3 Clinical Research Center, Changhai Hospital, Second Military Medical University, Shanghai, China; 4 Department of Sea-Air Examination Center, Navy General Hospital, Beijing, China; 5 Department of Laboratory Diagnosis, First Affiliated Hospital of Second Military Medical University, Shanghai, China; Cedars-Sinai Medical Center; UCLA School of Medicine, United States of America

## Abstract

Retinal neovascularization (RNV) is an eye disease that can cause retinal detachment and even lead to blindness. RNV mainly occurs in the elderly population. The pathogenesis of RNV has been previously reported to be highly related to the expression of vascular endothelial growth factor A (VEGFA), basic fibroblast growth factor (bFGF) and other angiogenic factors. It has also been reported that VEGFA and other factors associated with RNV could be regulated by certain microRNAs (miRNA), a group of small non-coding RNAs which are able to regulate the expression of many genes *in vivo*. Here, we demonstrate that the miRNA miR-410 is highly expressed in mice within two weeks after birth. miR-410 could suppress VEGFA expression through interaction with the 3′UTR of the VEGFA messenger RNA. Overexpressing a miR-410 mimic effectively suppresses VEGFA expression in various cell lines. Further experiments on oxygen-induced retinopathy (OIR) in mice revealed that eye drops containing large amounts of miR-410 efficiently downregulate VEGFA expression, prevent retinal angiogenesis and effectively treat RNV. These results not only show the underlying mechanism of how miR-410 targets VEGFA but also provide a potential treatment strategy for RNV that might be used in the near future.

## Introduction

Retinal neovascularization (RNV) is the formation of new blood vessels originating from the retinal veins and extending along the inner (vitreous) surface of the retina. RNV has been demonstrated to play a crucial role in several eye diseases such as diabetic retinopathy, retinal vein occlusion and retinopathy of prematurity [1], [2]. RNV can lead to vision-threatening complications such as vitreous hemorrhage, traction retinal detachment, neovascular glaucoma, and even blindness [3]–[6]. However, the pathological mechanism of RNV is not completely understood.

It has been proposed that a wide variety of angiogenic growth factors, such as vascular endothelial growth factor (VEGF) [7]–[9], insulin/insulin-like growth factor (IGF) [10], [11], and hypoxia-inducible factor-1 alpha (HIF-1A) [12], [13], may mediate RNV. Among these angiogenic growth factors, VEGF has been demonstrated to be a major contributor to neovascularization in the retina. Therefore, understanding the transcriptional regulation of VEGF may be conducive to exploring the role of transcriptional regulators in RNV.

MicroRNAs (miRNAs), small non-coding RNA molecules that suppress gene expression by interacting with the 3′ untranslated region (UTR) of target mRNAs, have also been linked to RNV [14], [15]. Recently, microarray analysis of miRNAs has shown that 177 miRNAs are highly expressed during the developmental process in many organs, such as ovary, of newborn mice [16]–[18]. Additionally, the developmental process of organs including ovary is closely related to neovascularization [19]–[21]. Therefore, we hypothesize that there may be some miRNAs that function in regulating neovascularization in the retinas of newborn mice. Recently, some reports have highlighted that miRNAs can inhibit neovascularization via regulating angiogenic growth factors including VEGFA and basic fibroblast growth factor (bFGF) [14], [22]–[24]. The expression of VEGFA has been demonstrated to be significantly suppressed by miRNAs [24]–[27]. Our correlation analysis by Targetscan on angiogenic growth factors and the 177 neonatally up-regulated miRNAs revealed three miRNAs, miR-410, miR-590-5p, and miR-200b, might play key roles in suppressing the expression of angiogenic factors in development (http://www.targetscan.org/). Previous studies also confirm the relationship between tumor vascularization and VEGFA overexpression [28], [29]. Therefore, we hypothesize that miR-410 might mediate a similar effect on VEGFA expression in RNV, given that tumor growth and RNV both share the trait of abnormal blood vessel growth closely associated with VEGFA overexpression. In this study, we further investigate the effect of miR-410 on RNV in a murine OIR model. We demonstrate that miR-410 suppresses VEGF expression and blocks OIR. Furthermore, this study indicates that VEGF inhibition by miR-410 may represent a novel therapeutic strategy to treat RNV in human patients.

## Materials and Methods

### Ethics statement

All animal experiments were conducted in accordance with the guidelines of the ARVO statement for the “Use of Animals in Ophthalmic and Vision Research” and were approved by the Ethics Committee of Changhai Hospital, Second Military Medical University.

### Employment of OIR mouse model

The neonatal mouse model of OIR was used as previously described [30], [31]. C57BL/6 mice were placed in a (75±2) % oxygen chamber at postnatal day 7 (P7), returned to room air at P12, and euthanized at P17.

### Plasmid preparation, identification and purification

The sequences of miR-410 and miR-26a were retrieved from the miRNA database miRBase (www.mirbase.org) (miR-410: AAUAUAACACAGAUGGCCUGU; miR-26a: TTCAAGTAATCCAGGATAGGCT; miR-mock: GAAATGTACTTGAGCGTGGAGAC.) and synthesized by Gene-Pharma (Promoter U6: CTGCGCAAGGATGACACGCAAA). The plasmid vector pLKO.1 (Sigma) with green fluorescein protein (GFP) report gene was used to construct the pLKO-miR-410 plasmid (Table 1). The construct result was confirmed by direct DNA sequencing. The control vector pLKO-mock and the irrelevant vector pLKO-miR-26a were used as controls. Plasmids pLKO-miR-410, pLKO-mock and pLKO-miR-26a were transformed in competent DH5α cells and extracted using the Plasmid Qiagen Midi Kit (Qiagen).

**Table 1:** Insert Sequences of recombinant pLKO plasmid.

miRNA-mimics	
**Name**	**Sequence(5′-→-3′)**
pLKO-miR-26a	UUCAAGUAAUCCAGGAUAGGCU
pLKO-mock	GAAATGTACTTGAGCGTGGAGAC
pLKO-miR-410	AAUAUAACACAGAUGGCCUGU

### Plasmid intravitreal injection or eye drop delivery

OIR mice and normal control mice at P12 were anesthetized through intraperitoneal injection of ketamine hydrochloride (40 mg/kg) and xylazine hydrochloride (10 mg/mL). The mice were then intravitreally injected with plasmids pLKO-mock, pLKO-miR-410 or pLKO-miR-26a. The OIR mice were given a 0.4 µl intraocular injection of 5 µg/µl pLKO-miR-410 or pLKO-mock in one eye. Intravitreal injection was performed by inserting a 32 gauge Hamilton needle (200 µm) into the vitreous body of anesthetized OIR mice at a site 1 mm posterior to the limbus of the eye. Insertion and infusion were performed and directly viewed through an operating microscope. Care was taken not to injure the lens or the retina. The tip of the needle was positioned over the optic disk and a volume of 0.4 µl was slowly injected into the vitreous. Any eyes that exhibited damage to the lens or retina were discarded and not used for analysis [32]. In addition, the plasmids were also condensed into eye drops and delivered to the eyes of mice (0.08 µg/µl, 10 µl per eye, six doses of eye drops three times a day) [33].

### Fluorescein angiography of retinal neovascularization

C57BL/6 mouse pups injected with plasmids pLKO-miR-410, pLKO-miR-26a and pLKO-mock were anesthetized at P17. The retinal vasculature was visualized by fluorescein angiography as previously reported [34]. Mice were perfused through the left ventricle with 10 mg/ml high-molecular weight fluorescein-dextran (Sigma) in PBS. Eyes were enucleated and fixed in darkness with cold 4% paraformaldehyde for 2 h. After removal of the lens, the retina was flat-mounted with glycerol–gelatin. The vasculature was analyzed by fluorescence microscopy.

### Detection of the presence of pLKO-miR-410

Retinas of treated mice were removed and washed with PBS three times in order to eliminate remaining blood and eye drops. DNA and RNA were extracted using TRIzol (Invitrogen). The primer set used to detect pLKO-miR-410 was designed using the U6 promoter (CTGCGCAAGGATGACACGCAAAT) as the upstream primer and the primer of miR-410 (ACAGGCCATCTGTGTTATATT) as the downstream primer.

### Retinal vascular endothelial cell proliferation

After OIR mice were sacrificed at P17, the eyeballs were obtained and the lenses were removed. Then, the eyeballs were fixed at 25°C for 48 h in Perfix (4% paraformaldehyde, 20% isopropanol, 2% trichloroacetic acid, 2% zinc chloride) and soaked in 70% Ethanol for 24 h. Ten fixed eyes from each group were then embedded in paraffin, and twenty 4-µm-thick sections were cut from both sides of the optic nerve. The sections were stained with hematoxylin-eosin (H&E) staining and observed under light microscopy. The thickness of the outer nuclear layer was represented by the average thickness 0.5 mm superior and inferior to the optic nerve head as described previously [34]. Proliferating retinal vascular endothelial cell nuclei were counted and analyzed.

### miRNA target search

Open-sourced software based on sequence complementarity, TargetScan 5.1 (www.targetscan.org), was used for miRNA target predictions.

### Cell culture

The human umbilical vein endothelial cells (HUVECs) and human retinal vascular endothelial cells (HRCECs) in our study were kindly gifted by Wenjing Zhang from Wisent Biotechnology Co., Ltd (Nanjing) [35]. Gifted cells were cultured in high-glucose DMEM (Corning) with 10% fetal bovine serum (Corning) with stimulus, ITS (Sigma) and vascular cell factor, β-ECGF (R&D). Suspended cells were removed during the following 72-hour culture. The cells were passaged after 72 hours. The second generation of the HRCECs was used in the following experiments. Both cell lines were transfected with plasmids pLKO-miR-410 and pLKO-miR-410-antisense using Lipofectamine 2000 (Life Tech) and cultured for 48 h.

### Quantitative real-time reverse transcription PCR assay

RNA was isolated from the HUVEC and HRCEC cell lines and the retina of OIR mice using a small RNA isolation kit (Ambion). Each PCR reaction was performed in triplicate in a 25 µl volume using SYBR Green AssayMasterMix (Applied Biosystems) for 3 min at 95°C, followed by 40 cycles of 95°C for 15 s, 60°C for 30 s and 72°C for 45 s in a Bio-Rad iCycler (Bio-Rad Laboratories)[36].

### Western blotting (WB)

The lysates of HUVECs, HRCECs or retinas were cleared by centrifugation at 12,000 g for 20 min at 4°C. The protein concentration of the lysate was determined using the BCA assay. Then, 20 µg (cell lines) or 5 µg (retina tissue from mice) of protein from the lysates was resolved by SDS-PAGE in 10% denaturing gels and transferred onto nitrocellulose membranes (Bio-Rad). Immunoblotting was performed by incubating the membranes with rabbit primary antibodies against VEGF (#19003-1-AP, Proteintech) (1:1000 in 5% nonfat milk powder/0.1% Tween-20 in PBS) overnight at 4°C. After incubating with horseradish peroxidase-conjugated secondary IgG antibody (Santa Cruz), the membranes were developed with chemiluminescence. The signal intensity was quantified by densitometry using software.

### Statistical analysis

Data are presented as the mean ± standard deviation (SD). The significance of differences was evaluated by t-test. Differences with P values <0.05 were considered statistically significant.

### Vascular density analysis

Tissue samples were fixed with neutral formalin, embedded with paraffin and sectioned. All samples were stained with DAPI and CD34-FITC (#AB8158, Abcam), and the resulting pictures were captured under a fluorescence microscope. After superimposing the target pictures, analysis was conducted using ImagePro 6.0, and the numbers of CD34^+^ and DAPI^+^ cells were counted. Relative vascular density was calculated by dividing the number of CD34^+^ cells by the number of DAPI^+^ cells. For each sample, five fields were randomly chosen to calculate the average vascular density.

### Chromatin immunoprecipitation experiment

Tissue samples were separated into single cell suspensions and ultrasonicated. After incubation with Protein A agarose (Millipore) to remove disturbed background, the samples were incubated with AGO1 antibody (#AB5070, Abcam). Protein A agarose was then used to precipitate the antigen-antibody complex. The negative control consisted of cells incubated with Protein A agarose but without AGO1 antibody. RNase Inhibitor (Promega) was used to protect the RNA throughout the above procedure. The precipitated complex was washed and RNA was extracted and reverse transcribed with an RNA kit (Promega). The mRNA sequences of VEGFA were detected with specific primers (Table 2).

**Table 2:** mRNA sequences of primers for VEGFA detection.

Primers for Detection	
**Name**	**Sequence (5′-→-3′)**
R-H-VEGFA-F	CTACCTCC ACCATGCCAAGT
R-H-VEGFA-R	TTTCTTGCGCTTTCGTTTTT
R-M-bFGF-F	GCCAACCGGTACCTTGCTAT
R-M-bFGF-R	GTCCCGTTTTGGATCCGAGT
R-M-TNF-F	CCAGACCCTCACACTCACAA
R-M-TNF-R	ATAGCAAATCGGCTGACGGT
R-M-PCNA-F	AGATGCCGTCGGGTGAATTT
R-M-PCNA-R	TGGTTACCGCCTCCTCTTCT
R-M-VEGFA-F	TATTCAGCGGACTCACCAGC
R-M-VEGFA-R	AACCAACCTCCTCAAACCGT
R-H-miR-410-F	AATATAACACAGATGGCCTGT

### Luciferase reporter gene assay

The mRNA sequence targeted by the miRNA was predicted using TargetScan. The sequence was synthesized and constructed into the reporter vector pmiRGLO (Promega) (Table 3). The pmiRGLO vector containing the predicted mRNA was then co-transfected with its corresponding miRNA into HUVEC and HRCEC. Should the miRNA indeed suppress gene expression by binding to the mRNA sequence on the pmiRGLO plasmid, the luminescence of firefly luciferase would decrease and the luminescence of Renilla luciferase would remain unchanged.

**Table 3:** Sequences of primers for recombinant pmiRGLO plasmid.

Primers for Molecule Clone	
**Name**	**Sequence (5′-→-3′)**
C3-H-VF-F	GATCGTTTAAACACACACCCACCCACATACAT
C3-H-VF-R	GATCTCTAGAGAATATATATATTTTATATA
C3-H-V1-F	TCGAGAGAGAAAGTGTTTTATATACA
C3-H-V1-R	AGCTTGTATATAAAACACTTTCTCTC
C3-H-V1M-F	TCGAGAGAGAAAGTGTTTGCGCGCCA
C3-H-V1M-R	AGCTTGGCGCGCAAACACTTTCTCTC

## Results

### OIR model in the mouse

An OIR mouse model was employed to explore the pathogenesis of RNV. As expected, neovascularization in the eyes of the OIR mouse model was significantly increased compared with eyes in the control group (Fig S1a). Fluorescein angiography showed that more RNV and larger tracts of non-perfused areas were present in the eyes of mice from the OIR group than in the eyes of mice from the control group (Fig S1b). In addition, histological analysis indicated that the number of preretinal neovascular nuclei (PRNN) was increased in the OIR group compared to the control group. The PRNN number of the OIR group was 1.39±1.12, 16.67±3.51, 42.31±4.69, 39.23±4.5, and 0.93±0.85 at P13, P15, P17, P19, and P23, respectively. In comparison, the number of PRNN in the control group was 0.96±0.91, 0.94±0.92, 0.89±0.91, 0.9±0.89, and 0.89±0.97 at P13, P15, P17, P19, and P23, respectively (Fig S1c).

### Expression of VEGFA is up-regulated in OIR mice

To investigate the underlying mechanism(s) leading to RNV in mice, real-time PCR analysis was performed to determine levels of common angiogenic factors such as VEGF, bFGF, and TNFA in retinal tissues of OIR mice (P17) [37], [38]. Compared to the retinal tissue of the control group, the expression of several angiogenic factors, especially VEGFA, was significantly highly expressed in retinal tissues of OIR mice (Fig S1d). Further WB analysis confirmed that VEGFA was more highly expressed in the retinal tissues of OIR mice than of control mice (Fig S1e). It is widely acknowledged that high expression of VEGFA in tissues could result in vascularization [39]. Therefore, we selected VEGFA as the main focus of our follow-up studies.

During embryonic developmental stages, high expression of VEGFA is observed throughout the mouse body. In most tissues, including the retina, high VEGFA expression does not persist after birth. Previous studies have indicated that sharp changes in miRNA expression occur before and after birth in mice, and these changes seem to be involved in modulating body functions. Therefore, we hypothesize that one or several miRNAs may be involved in regulating VEGFA expression.

### 3′UTR of VEGFA is a potential target of miRNAs

**Figure 1:**
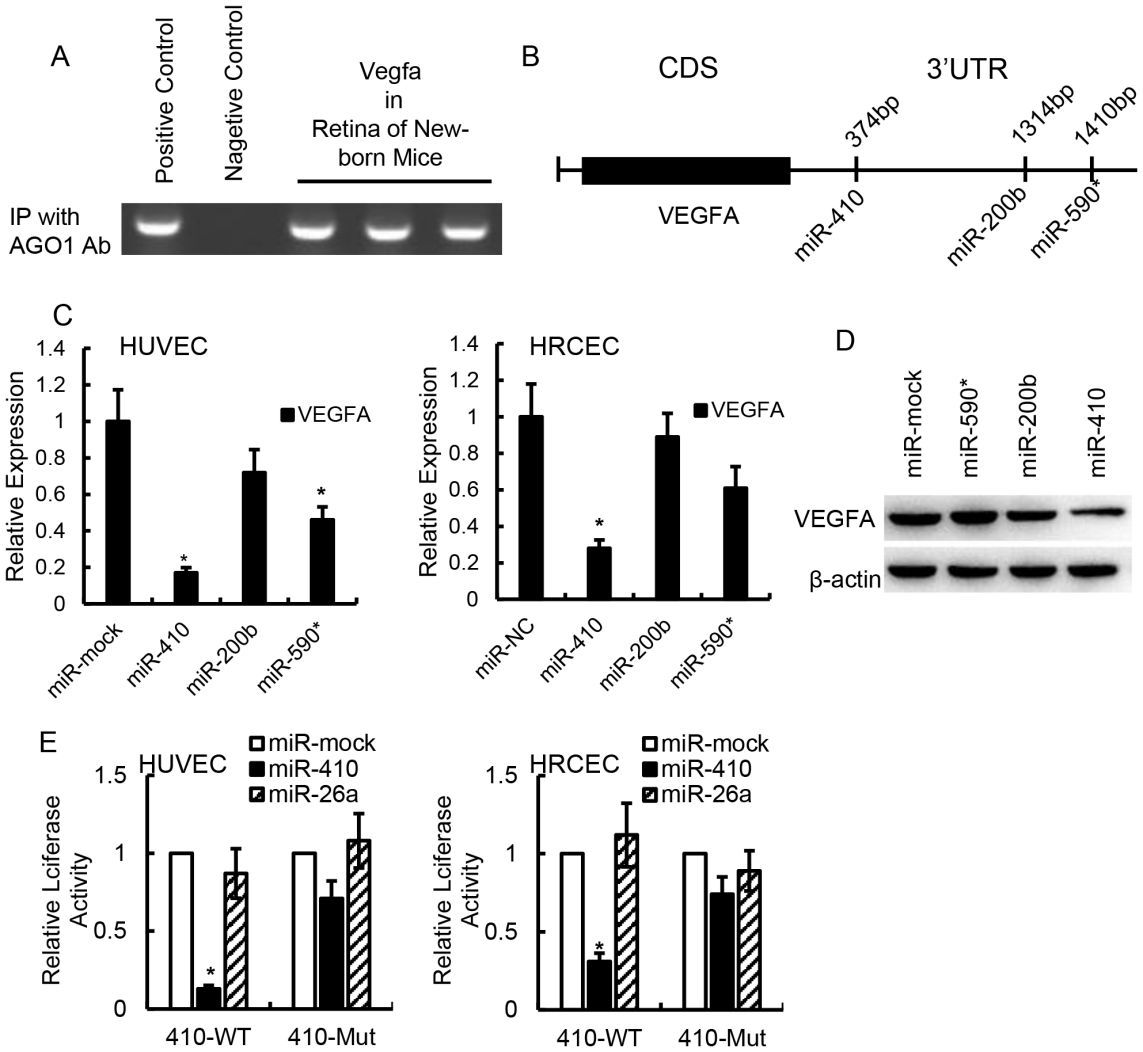
miR-410 suppresses VEGFA expression through binding to the 3′UTR of VEGFA mRNA. A. Co-immunoprecipitation assay for VEGFA in the retinas of newborn mice. VEGFA mRNA in the retinas of newborn mice bound to AGO1, the core component of RISC, indicating a regulatory role of miRNAs in VEGFA expression in these tissues. **B**. Bioinformatic analysis of the VEGFA mRNA sequence. Three miRNAs were specifically complementary to the 3′UTR of VEGFA mRNA among 177 miRNAs that were highly expressed in newborn mice. **C**. qPCR analysis for VEGFA expression after the three miRNAs mimics were transfected into HUVECs and HRCECs. VEGFA expression was suppressed by miR-410 at the molecular level compared with miR-200b and miR-590-5p transfection. *P<0.05 **D**. Western blot assay for VEGFA expression after the three miRNAs mimics were transfected into HUVECs and HRCECs. VEGFA expression was suppressed by miR-410 at the protein level. **E**. Luciferase reporter gene experiment on HUVECs and HRCECs after transfection with miR-410 and miR-410-Mut. Luminescence of the reporter gene in cells transfected with miR-410 was much lower compared with the mutated group. *P<0.05.

In order to address whether a miRNA can down-regulate VEGFA expression, we used chromatin immunoprecipitation to detect whether VEGFA combined with miRNA functional molecules. It has been reported that miRNA-mediated degradation of mRNA is regulated by the RNA-induced silencing complex (RISC), an AGO-cored protein family [40], [41]. Previous studies have reported that AGO1 is a main component of RISC, while AGO2 is one of a number of proteins that load miRNA molecules into RISC [40]–[42]. Therefore, an antibody of AGO1 was chosen to capture RISC and detect RNA associated with it. Indeed, mRNA of VEGFA was found in RISC, which suggests that VEGFA is regulated by RISC and miRNA (Fig 1a).

Given the above results, we performed further bioinformatic analysis on VEGFA mRNA. As shown by Targetscan, among 177 miRNAs that are specifically highly expressed in newborn mice, three miRNAs might target the 3′UTR of VEGFA [16]. These three miRNAs were miR-410, miR-590-5p and miR-200b (Fig 1b).

### miR-410 reduces VEGFA expression in HUVEC and HRCEC

To determine which of the three miRNAs could effectively suppress VEGFA expression, we synthesized mimics of miR-410, miR-590-5p and miR-200b (Table 4) and transfected them into human umbilical vein endothelial cells (HUVECs) and retinal vascular endothelial cells (HRCECs), cells in which VEGFA is naturally highly expressed. Expression of VEGFA was significantly down-regulated in both cell lines transfected with these three miRNAs, compared with cells transfected with miR-mock and an irrelevant miRNA, miR-26a. Among the three neonatally up-regulated miRNAs predicted to interact with VEGFA mRNA, VEGFA expression was reduced furthest after miR-410 transfection (Fig 1c).

**Table 4:** Sequences of miRNA-mimics.

miRNA-mimics	
**Name**	**Sequence(5′-→-3′)**
miR-26a	UUCAAGUAAUCCAGGAUAGGCU
miR-200b	UAAUACUGCCUGGUAAUGAUGA
miR-590-5p	GAGCUUAUUCAUAAAAGUGCAG
miR-mock	GAAATGTACTTGAGCGTGGAGAC
miR-410	AAUAUAACACAGAUGGCCUGU

Further WB analysis showed VEGFA expression was significantly reduced at the protein level after transfection with miR-410 in both HUVECs and HRCECs. These results indicate miR-410 could efficiently suppress VEGFA expression in HUVECs and HRCECs (inhibition efficiency: 88% and 81%, respectively) (Fig 1d).

### miR-410 suppresses VEGFA through binding to the 3′UTR of VEGFA mRNA

Further analysis of the miR-410 sequence revealed multiple binding sites on the 3′UTR of VEGFA mRNA. Ten base pairs of the miR-410 sequence were fully complementary to sequences in the VEGFA mRNA, and the site at 374 bp of the miR-410 sequence bound the most firmly to 3′UTR of VEGFA. To investigate the effects of miR-410 on VEGFA expression, a pmiRGLO plasmid with a 60 bp sequence (359-419) from the VEGFA mRNA 3′UTR and a luciferase reporter gene was constructed and then co-transfected with miR-410 into HUVECs and HRCECs. Compared to cells transfected with miR-mock and miR-26a, luminescence of the reporter gene in cells transfected with miR-410 was much lower, which indicates that miR-410 may bind to the 3′UTR (359∼419) of VEGFA to suppress its expression (Fig 1e left).

To further verify the previous finding, we mutated the miR-410 binding site in the 3′UTR of VEGFA (site 374–382 in the 3′UTR) and repeated the previous transfection experiment. The result showed no significant reduction of luminescence from the reporter gene (Fig 1e right). This confirms that miR-410 inhibits VEGFA expression through binding to locus 374–389 of the VEGFA mRNA 3′UTR.

### Suppression of VEGFA by miR-410 has therapeutic potential for RNV in an OIR mouse model

**Figure 2:**
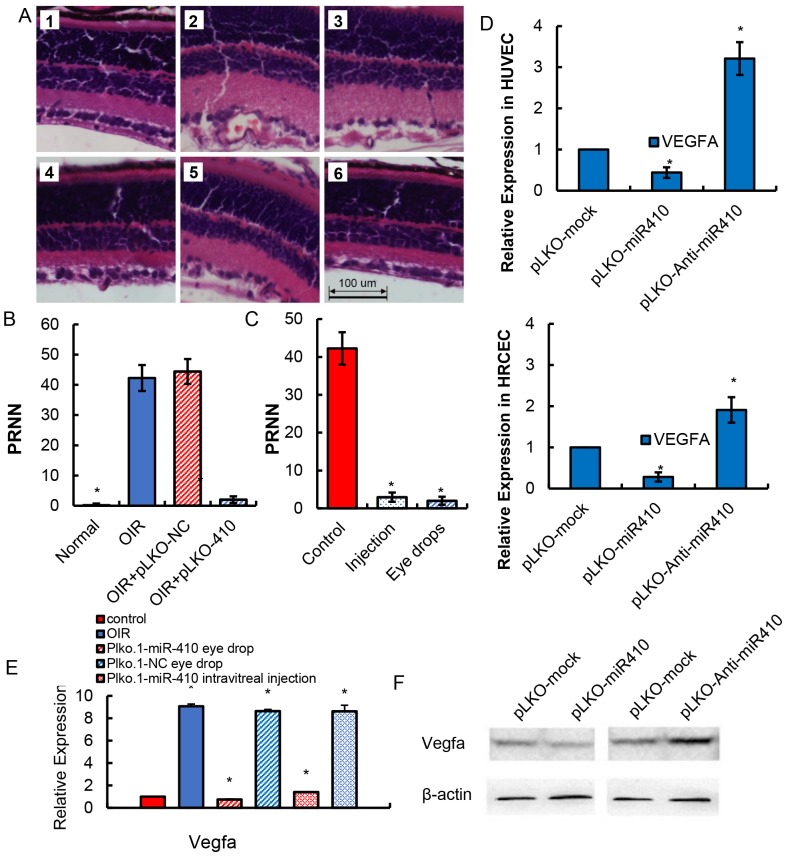
Overexpression of miR-410 efficiently inhibits neovascularization in mice retinas by effectively suppressing VEGFA expression. A. HE staining of proliferative neovascularization in murine retinal tissues. **1**: control mice; **2**: OIR mice; **3**: OIR mice with pLKO-mock intravitreal injection; **4**: OIR mice with pLKO-miR-410 intravitreal injection; **5**: OIR mice with pLKO-mock eye drops; **6**: OIR mice with pLKO-miR-410 eye drops. miR-410 administration either through direct intravitreal injection or eye drops effectively inhibits retinal revascularization. **B**. Statistical analysis of PRNN showed that administering eye drops containing pLKO-miR-410 to OIR mice could effectively inhibit retinal neovascularization (Left panel). **C**. Local intravitreal injection of pLKO-miR-410 plasmid directly into OIR mice also led to a trend towards decreased neovascularization. However, no significant difference was observed. **D**. qPCR analysis for VEGFA expression in both HUVECs and HRCECs before and after miR-410 interference by siRNA. VEGFA mRNA was significantly down-regulated with miR410 overexpression and up-regulated with miR-410 interference compared with controls. *P<0.05 **E**. qPCR analysis for VEGFA expression in murine retinas before and after miR-410 interference by siRNA. VEGFA mRNA was significantly down-regulated with miR410 overexpression and up-regulated with miR-410 interference compared with controls. *P<0.05 **F**. Western blot assay for VEGFA expression in murine retinas before and after miR-410 interference. Protein levels of VEGFA were also down-regulated with miR410 overexpression and up-regulated with miR-410 interference.

To assess the therapeutic potential of miR-410 in treating RNV, we constructed a plasmid to express a miR-410 mimic (pLKO-miR-410), a plasmid to express an interfering miR410-antisense product (pLKO-miR-410-Anti), a control plasmid expressing an irrelevant miRNA (pLKO-miR-26a) and a control plasmid encoding a mock sequence (pLKO-mock). The plasmid solutions were processed into eye drops to treat OIR mice. First, to determine whether the therapeutic plasmid could reach the target retinal tissue, we detected the presence of pLKO-miR-410 in retinas of OIR mice at the molecular level. Analysis by qPCR revealed that the plasmid was present in the retina (Fig S2a). HE staining revealed that the number of retinal neovascules dropped significantly in OIR mice treated with the pLKO-miR-410 plasmid (Fig 2a). Statistical analysis on the number of preretinal neovascular nuclei (PRNN) of vascular endothelial cells which broke across the internal limiting membrane of the retina revealed that eye drops containing pLKO-miR-410 could reduce the PRNN number back to the level seen in non-OIR control mice (Fig 2b). Moreover, local intravitreal injection of the pLKO-miR-410 plasmid into OIR mice yielded almost the same outcome as application of eye drops containing the same plasmid to these mice, both methods of administration effectively suppressing neovascularization (Fig 2c). Further analysis at the molecular level revealed that VEGFA mRNA was down-regulated with miR410 overexpression (inhibition efficiency: 90%) (Fig 2e, Fig 2d middle) and up-regulated with miR-410 interference (Fig 2d right). Additional WB analysis revealed a similar trend at the protein level (Fig 2f). Apart from investigating the expression level of VEGFA, we also detected the expression level of other angiogenic factors, namely, FGF-2 and PLGF. Analysis by qPCR showed that there was no significant drop in the expression of these factors (Fig S2b). A luciferase reporter gene experiment on HUVEC and HRCEC further confirmed that miR-410 does not suppress FGF-2 and PLGF. Thus, miR-410 might exclusively target VEGFA (Fig S2c). These results further proved that VEGFA expression could be effectively suppressed by miR-410, thus preventing RNV to some extent.

## Discussion

In this study, we focused on the role of miR-410 in RNV. We employed an oxygen-induced RNV mouse model and confirmed that VEGF was significantly increased in retinas from OIR mice. Overexpression of miR-410 in the retina effectively prohibits the high levels of VEGF in the OIR model and thus significantly reduces RNV in this model. To our knowledge, this is the first comprehensive study on the role of miR-410 in RNV.

MicroRNAs are a class of 22-nucleotide RNAs that do not code proteins. Instead, miRNAs bind to target mRNAs to prevent protein translation and play a key role in a wide range of physiological and pathological processes [43]-[46]. MicroRNAs are ubiquitously expressed in plants and animals and constitute an essential component of gene regulation [47]–[50]. Karali et al. demonstrated that engineering miRNA target sites into the 3′UTR of virally delivered transgenes could effectively modify the pattern of transgene expression in the mammalian retina [51]. Recent studies show that some miRNAs play a crucial role in RNV. For example, microarray analysis demonstrates that miR-106a, -146, -181, -199a, -214, -424, and -451 are substantially increased and miR-31, -150, and -184 are substantially decreased in ischemic retinas. Intraocular injection of pre-miR-31, -150, or -184 significantly reduces ischemia-induced RNV, and injection of pre-miR-31 or -150 also significantly reduced choroidal neovascularization [14]. In our previous works, we found miR-410 could reduce neovascularization in cancer tissues. However, the role of miR-410 in RNV remains to be elucidated. In this study, we investigated the effect of miR-410 on RNV in OIR mice. As expected, miR-410 intravitreal injection reduced RNV in the OIR model. This result indicates that miR-410 is also an important regulator of RNV. Based on this, the mechanisms underlying the effect of miR-410 on inhibiting RNV were further investigated.

It is now apparent that VEGF plays a critical role both in retinal vascular development [52] and in pathologic angiogenesis in ischemic retinopathies and other forms of ocular neovascularization [53]. VEGF has been documented to be highly expressed in models of these processes [54]–[56]. In the present study, we employed an oxygen-induced RNV mouse model and also found a high expression level of VEGF. Intravitreal injection of VEGF induces the retinal vascular changes that occur in experimental diabetes, including retinal leukostasis and concomitant BRB breakdown, whereas blockade of VEGF abolishes retinal leukostasis and vascular leakage [57], [58]. Recent studies also show that VEGF signaling could be regulated by miRNAs [25], [27], [59]. Intravitreal injection of miR-126 overcomes the high levels of VEGF through downregulating p38 and ERK signaling molecules in an OIR model, and thus reduces retinal neovascularization in this model [14]. In this study, we found that VEGF was a target gene of miR-410 by bioinformatics prediction, and miR-410's ability to regulate VEGF expression was further confirmed in *in vitro* experiments. miR-410 inhibits VEGFA in endothelial cells and presumably has similar activity in other cells in the retina in order to mediate the large reduction in VEGF production. Also, the expression of VEGF in the retina was significantly suppressed after miR-410 intravitreal injection. This suggests that miR-410 might inhibit RNV in OIR mice by down-regulating VEGF.

Many groups have turned to the retina as a model system for studying miRNA gene regulation [60]. Gene therapies for neovascular age-related macular degeneration as well as retinoblastoma are currently undergoing clinical trials [61], [62]. The findings in the present study provide evidence to show the important roles of miRNAs. Because the signaling pathways modulating miR-410 biogenesis are still unknown, further investigation into related mechanisms are important in developing new therapies for preventing RNV.

It is surprising that miR-410 eye drop delivery could inhibit RNV. It was recently reported that exogenous plant miRNAs are present in the sera and tissues of various animals and that these exogenous plant miRNAs can regulate the expression of target genes involved in the physiology of mammals [63]. There may be an undiscovered mechanism related to miRNAs to explain these puzzling results. It is possible that miRNAs in eye drops first transfect into corneal and/or sclera cells and then affect other ocular tissues by paracrine action.

In summary, miR-410 was identified as a novel regulator of RNV acting most likely through down-regulating VEGF expression. Eye drops containing miR410 could effectively inhibit RNV in an OIR model. Revelation of such novel mechanisms helps us better understand the pathogenesis of eye diseases and will eventually be helpful in developing novel treatments.

## Supporting Information

Figure S1 **High expression of VEGFA in the OIR model.** A. HE staining of proliferative neovascularization in endothelial cells from the retinas of OIR mice. **1**: normal mice; **2**: OIR mice, P13; **3**: OIR mice, P15; **4**: OIR mice, P17; **5**: OIR mice, P19; **6**: OIR mice, P23; (Retinal neovascules are indicated by arrows) **B**. Fluorescein angiography of the OIR mouse model. In OIR groups, there were more retinal neovascularization and large tracts of non-perfused areas. (Retinal neovascules are indicated by arrows) **C**. Statistical analysis. A significant increase in the number of neovascules in OIR mouse model was observed when compared to control mice. The number of PRNN of vascular endothelial cells which broke across the internal limiting membrane of the retina increased significantly under hyperoxia-induction. *P<0.05, compared with control mice. **D**. qPCR analysis for expression of common angiogenic factors. Of the factors tested, VEGFA expressed highest in retinal tissue. **E**. Western blot assay for VEGFA expression in the retinas of control and OIR mice. **PRNN**: preretinal neovascular nuclei; **PRE**: retinal pigment epithelium; **ONL**: outer nuclear layer; **INL**: inner nuclear layer; **GCL**, ganglion cell layer.
(TIF)

Figure S2 **miR-410 might exclusively target VEGFA.** A. qPCR analysis for pLKO-miR-410 expression in retinas of OIR mice. *P<0.05 compared with controls. pLKO-miR-410 was observed in the retinas. **B**. The expression of the angiogenic factors FGF-2 and PLGF was also observed. No significant reduction of expression was found in FGF-2 and PLGF upon miR-410 overexpression. **C**. 3′UTRs of VEGFA, FGF2 and PLGF mRNAs were packaged into reporter gene pmiRGLO. Luciferase reporter gene experiments on HUVEC after cells were transfected with miR-410, miR-181 or miR-mock. Lower luminescence of the reporter gene in cells transfected with miR-410 compared with cells transfected with the mutated miRNA indicated that miR-410 specifically targets VEGFA. *P<0.05.
(TIF)
