# Supplementary figures and images for: MicroRNA-410 Reduces the Expression of Vascular Endothelial Growth Factor and Inhibits Oxygen-Induced Retinal Neovascularization
Source: PLoS One. 2014 Apr 28;9(4):e95665. doi: 10.1371/journal.pone.0095665 (PMC4002426; doi:10.1371/journal.pone.0095665)

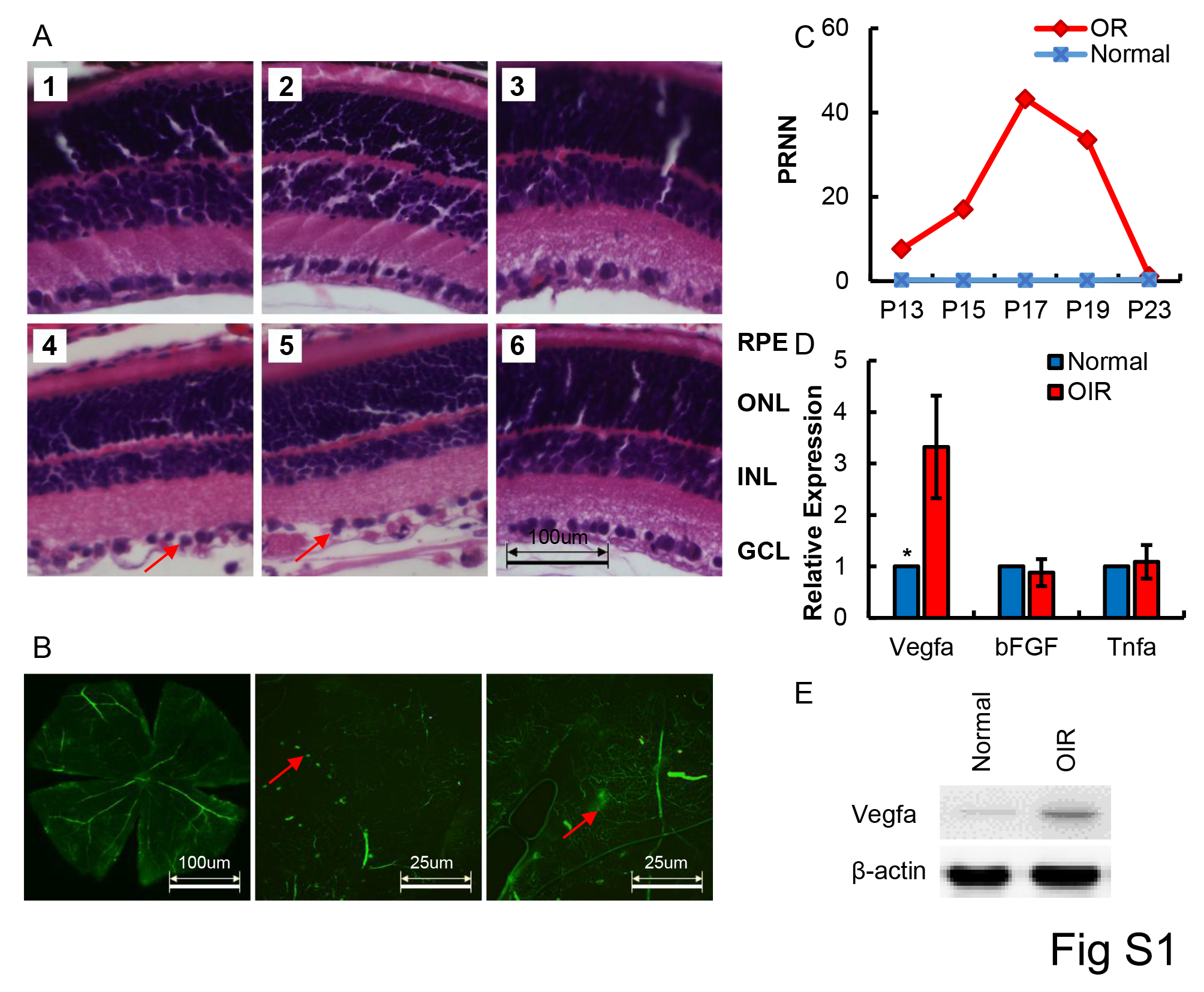

Supplement: Figure S1 — High expression of VEGFA in the OIR model. A. HE staining of proliferative neovascularization in endothelial cells from the retinas of OIR mice. 1: normal mice; 2: OIR mice, P13; 3: OIR mice, P15; 4: OIR mice, P17; 5: OIR mice, P19; 6: OIR mice, P23; (Retinal neovascules are indicated by arrows) B. Fluorescein angiography of the OIR mouse model. In OIR groups, there were more retinal neovascularization and large tracts of non-perfused areas. (Retinal neovascules are indicated by arrows) C. Statistical analysis. A significant increase in the number of neovascules in OIR mouse model was observed when compared to control mice. The number of PRNN of vascular endothelial cells which broke across the internal limiting membrane of the retina increased significantly under hyperoxia-induction. *P<0.05, compared with control mice. D. qPCR analysis for expression of common angiogenic factors. Of the factors tested, VEGFA expressed highest in retinal tissue. E. Western blot assay for VEGFA expression in the retinas of control and OIR mice. PRNN: preretinal neovascular nuclei; PRE: retinal pigment epithelium; ONL: outer nuclear layer; INL: inner nuclear layer; GCL, ganglion cell layer. (TIF) [file pone.0095665.s001.tif]

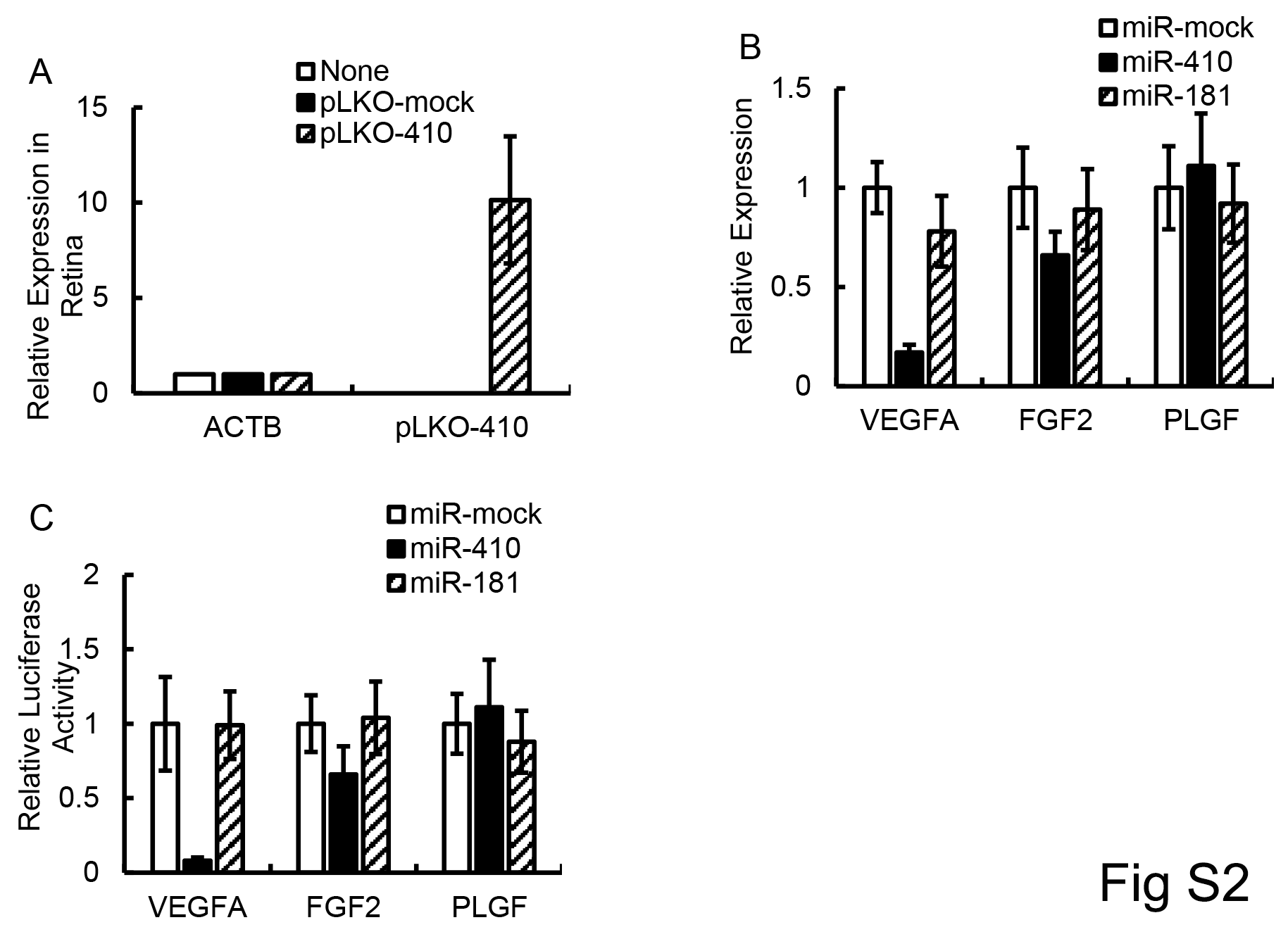

Supplement: Figure S2 — miR-410 might exclusively target VEGFA. A. qPCR analysis for pLKO-miR-410 expression in retinas of OIR mice. *P<0.05 compared with controls. pLKO-miR-410 was observed in the retinas. B. The expression of the angiogenic factors FGF-2 and PLGF was also observed. No significant reduction of expression was found in FGF-2 and PLGF upon miR-410 overexpression. C. 3′UTRs of VEGFA, FGF2 and PLGF mRNAs were packaged into reporter gene pmiRGLO. Luciferase reporter gene experiments on HUVEC after cells were transfected with miR-410, miR-181 or miR-mock. Lower luminescence of the reporter gene in cells transfected with miR-410 compared with cells transfected with the mutated miRNA indicated that miR-410 specifically targets VEGFA. *P<0.05. (TIF) [file pone.0095665.s002.tif]
